# Identifying Priority Areas for Conservation: A Global Assessment for Forest-Dependent Birds

**DOI:** 10.1371/journal.pone.0029080

**Published:** 2011-12-19

**Authors:** Graeme M. Buchanan, Paul F. Donald, Stuart H. M. Butchart

**Affiliations:** 1 The Royal Society for the Protection of Birds, Edinburgh, United Kingdom; 2 The Royal Society for the Protection of Birds, Sandy, Bedfordshire, United Kingdom; 3 BirdLife International, Cambridge, United Kingdom; University of Georgia, United States of America

## Abstract

Limited resources are available to address the world's growing environmental problems, requiring conservationists to identify priority sites for action. Using new distribution maps for all of the world's forest-dependent birds (60.6% of all bird species), we quantify the contribution of remaining forest to conserving global avian biodiversity. For each of the world's partly or wholly forested 5-km cells, we estimated an impact score of its contribution to the distribution of all the forest bird species estimated to occur within it, and so is proportional to the impact on the conservation status of the world's forest-dependent birds were the forest it contains lost. The distribution of scores was highly skewed, a very small proportion of cells having scores several orders of magnitude above the global mean. Ecoregions containing the highest values of this score included relatively species-poor islands such as Hawaii and Palau, the relatively species-rich islands of Indonesia and the Philippines, and the megadiverse Atlantic Forests and northern Andes of South America. Ecoregions with high impact scores and high deforestation rates (2000–2005) included montane forests in Cameroon and the Eastern Arc of Tanzania, although deforestation data were not available for all ecoregions. Ecoregions with high impact scores, high rates of recent deforestation and low coverage by the protected area network included Indonesia's Seram rain forests and the moist forests of Trinidad and Tobago. Key sites in these ecoregions represent some of the most urgent priorities for expansion of the global protected areas network to meet Convention on Biological Diversity targets to increase the proportion of land formally protected to 17% by 2020. Areas with high impact scores, rapid deforestation, low protection and high carbon storage values may represent significant opportunities for both biodiversity conservation and climate change mitigation, for example through Reducing Emissions from Deforestation and Forest Degradation (REDD+) initiatives.

## Introduction

Enormous and growing environmental problems and a chronic shortage of resources to tackle them require conservationists to set priorities for investment [1], [2], [3]. Several global conservation prioritisation exercises have been undertaken, using a range of different criteria, primarily relating to biological importance and levels of threat [4], [5], [6]. They range in scale from large regions such as Biodiversity Hotspots [7], [8] to discrete sites such as Alliance for Zero Extinction sites [9], Important Bird Areas [10] and other Key Biodiversity Areas [11]. The ultimate goal of prioritisation exercises is to facilitate the safeguarding of the most important sites. This is often achieved through legislative means by designation as protected areas. However, the protected area network is far from complete [12], captures poorly the ranges of threatened species [13], [14], and is uneven in its coverage of different habitats [15], including different types of forests [15].

Identification of priority areas has hitherto resulted in binary classifications (each point on the planet's surface falls either inside or outside a particular set of sites), although a continuous score could be more informative in setting priorities and making comparisons within and outside such areas. We used a newly available dataset on the distributions of all bird species and maps of forest extent and loss to develop a continuous spatial score of conservation importance in order to help expand and augment existing conservation and protected area networks. The score for each cell is calculated as the sum (across the species mapped as present within the cell) of the inverse of the number of cells each of those species' distribution covers, and represents a measure of the contribution of that cell to the distributions of the species it contains [16], [17]. This measure is repeatable over time, and would permit the relative spatial comparison of scores with values from other taxa assessed in a similar manner. We focused on this class of organisms because distribution maps are available for all bird species and because birds are useful indictors for broader biodiversity [10]. We focused on forest because most of the planet's terrestrial biodiversity, especially its threatened biodiversity, is found in this habitat, including well over half of all bird species [18], and because the distribution and loss of forests are readily and precisely derived from remote sensing imagery.

Threat is an important consideration in conservation planning [19], so to assess which of the areas of highest importance for the world's forest bird species are particularly threatened we intersected the impact scores with spatial data on rates of recent deforestation and the distribution of protected areas. We then identified regions of high importance and threat that have least protection. Important Bird Areas (IBAs), are a global network of c.10,000 sites for the conservation of birds and other biodiversity identified using globally standardised quantitative criteria [10]. Intersecting these with the impact score highlighted a priority suite of clearly demarcated sites that are amenable to management for conservation. This assessment is timely in light of the commitment made in 2010 by the world's governments to expand the protected area network from 12% to 17% of land area by 2020, covering ‘especially areas of particular importance for biodiversity’ [20].

Finally, we considered the relevance of these results to the emerging REDD (Reducing Emissions from Deforestation and Forest Degradation) initiative, which aims to use market incentives to reduce greenhouse gas emissions by paying for avoided deforestation [21], [22]. We overlaid a global map of carbon stocks onto the impact scores, deforestation data and protected areas data to identify those areas of highest importance for forest birds that are most threatened, least protected and have high carbon stocks. These areas are arguably among the most urgent priorities for REDD+ and will deliver the greatest benefits to biodiversity [21], [23].

## Results

### The distribution of impact scores

**Figure 1:**
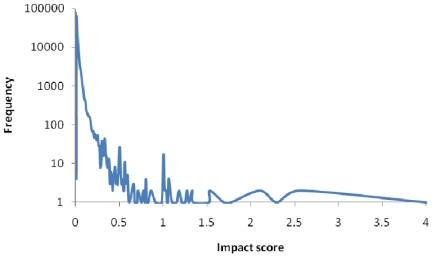
Frequency distribution (log scale) of 5-km cell impact scores. Plot smoothed to aid visual interpretation.

**Figure 2:**
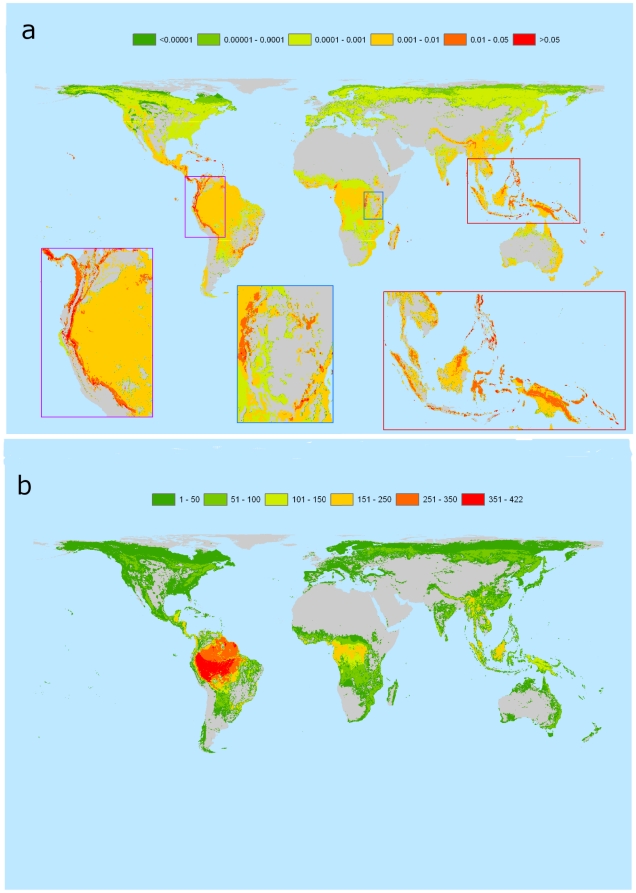
Impact scores (a) and forest bird species richness (b) in each of the world's 2.2 million forested 5-km grid squares. Areas in grey not forested.

**Figure 3:**
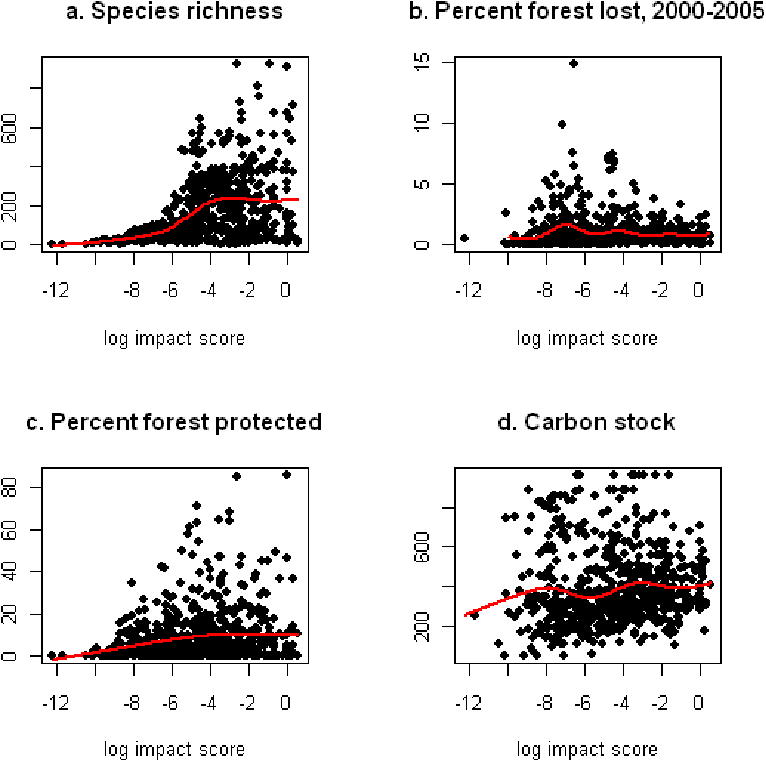
Scatterplots for each ecoregion of maximum impact score and (a) total forest bird species richness, (b) percent loss of forest during 2000–2005, (c) percent forest in protected areas and (d) carbon stocks (averaged across only forested cells in each ecoregions). Red lines indicate fitted GAMs.

Across the world's 2.2 million forested 5-km cells, impact scores ranged from just over zero in boreal tundra to a maximum of 4.01 in Hawaii's tropical moist forests. The average score was 0.0026±0.0000076 but the distribution was strongly skewed (Figure 1). The 1% of 5-km cells with the highest impact scores accounted for 27.2% of the sum of impact scores across all cells. Among the regions containing the highest scores were the Hawaiian islands, São Tomé and Príncipe, the islands of Indonesia, the Philippines and New Guinea, the Atlantic Forests and northern Andes of South America, while the lowest values were in arctic and arid ecoregions that contained small areas of forest (Figures 2a and S1, Tables 1 and S1). In contrast, the areas of highest bird species richness fell predominantly within the Amazon and Congo Basins (Figure 2b). The highest impact scores were associated with species-poor areas containing species with small ranges (Figure S2) and there was no simple relationship across all cells between impact score and bird species richness (Figures 2a, 2b, 3a, S3a). The 20 ecoregions with the highest impact scores within each biogeographic realm were generally islands, coastal areas and mountainous areas, although in the Afrotropics and the Neotropics extensive inland lowlands were also included (e.g. Cerrado in the Neotropics, Miombo in the Afrotropics) (Figure 2a, Table S1).

**Table 1:** The top 20 ecoregions ranked by maximum impact score with degree of recent forest loss, coverage of forest by protected areas and coverage by IBAs.

Ecoregion	Realm	Forest area (km^2^)	Max impact score	Mean % forest loss 2000–2005	% forest in PAs	% forest in IBAs
**1** Hawaii tropical forests	OC	4975	4.01	0.22	12.06	No data
**2** Palau tropical moist forests	OC	200	2.55	No data	0	50
**3** São Tomé, Príncipe and Annobon moist lowland forests	AT	850	1.78	No data	0	26.47
**4** Mascarene forests	AT	1150	1.69	0.09	10.87	30.43
**5** *Cordillera La Costa montane forests*	NT	12975	1.34	1.78	36.80	38.54
**6** *Northwestern Andean montane forests*	NT	48175	1.33	0.34	14.32	33.63
**7** *Brigalow tropical savanna*	AA	134725	1.33	0.46	4.81	0.52
**8** Jamaican moist forests	NT	4550	1.30	0.16	13.19	34.62
**9** *Chocó-Darién forests*	NT	67275	1.27	0.61	2.34	10.74
**10** *Pernambuco coastal forests*	NT	400	1.25	No data	0	18.75
**11** *Magdalena Valley montane forests*	NT	51475	1.19	0.38	2.72	23.85
**12** Fiji tropical moist forests	OC	9000	1.19	0.09	0	28.89
**13** Windward Islands forests	NT	1525	1.16	No data	0	22.95
**14** Sumba deciduous forests	AA	750	1.08	No data	0	53.33
**15** Halmahera rain forests	AA	25850	1.07	1.24	0.48	11.12
**16** Solomon Islands rain forests	AA	33850	1.06	0.28	0	No data
**17** *Serra do Mar coastal forests*	NT	56875	1.04	0.53	10.64	33.54
1**8** Rakiura Island temperate forests	AA	875	1.03	No data	85.71	No data
**19** *Mount Cameroon and Bioko montane forests*	AT	1175	1.02	2.40	10.64	76.6
**20** *Peruvian Yungas*	NT	115950	1.02	0.18	11	26.5

Italics indicate non-island ecoregions. Realms: AA Australasia, AT Afrotropics, NT Neotropics, OC Oceania.

### Recent forest loss

**Figure 4:**
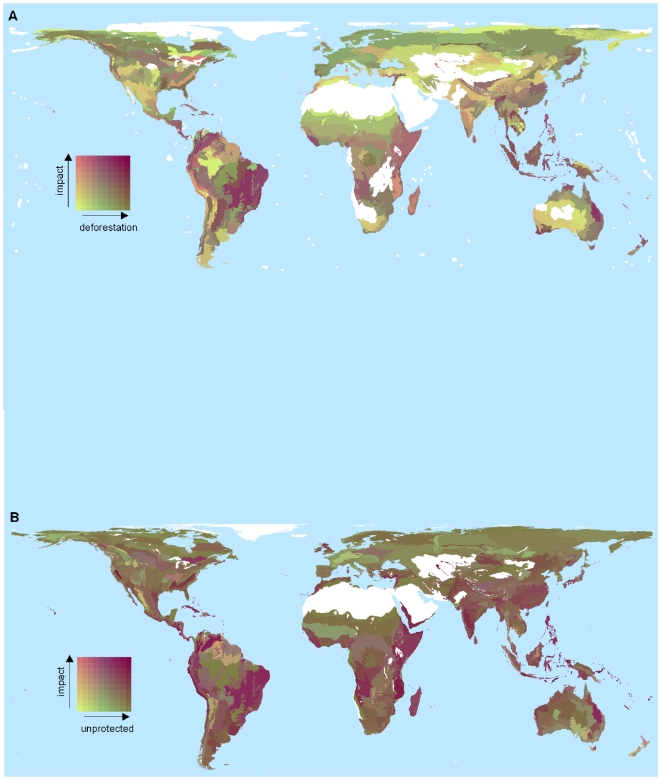
Bivariate plots showing ecoregion-level impact score and (a) percent loss of forest during 2000–2005, and (b) percent forest unprotected. Areas in white are non-forested ecoregions or lack data for one or both variables.

The 18-km squares assessed for forest loss in 2000–2005 by Hansen et al. [24] overlapped with 2,083,034 (94.6%) of the 5-km forested squares, but forest loss data were not available for some ecoregions containing high impact scores (e.g. Palau tropical moist forests and Pernambuco coastal forests; Tables 1, S2). For those ecoregions with data on forest loss, mean deforestation between 2000 and 2005 was 1.3%±0.0022. Ecoregions with the highest maximum scores for impact did not suffer disproportionately high rates of recent deforestation (Figure 3b), a pattern consistent across biogeographic realms (Figure S3b). However, a number of ecoregions had both high impact scores and high rates of recent deforestation including: Cordillera La Costa montane forests in the Neotropics, Halmahera rain forests in the Indo-malayan realm, Mount Cameroon and Bioko montane forests, and the Eastern Arc forests in the Afrotropics (Figure 4a).

### Protected areas and Important Bird Areas (IBAs)

The protected area network (which covers approximately 13% of the planet's land surface) encompasses 9% of forested 5-km cells. At a global scale there was a weak tendency towards greater protected area coverage in ecoregions with higher impact scores (Figures 3c, 4b), although patterns differed between biogeographic realms (Figure S3c). Forested 5-km cells falling within protected areas had impact scores approximately twice as large as those of squares outside protected areas (Figure S4). However, seven of the 20 ecoregions with the highest impact scores did not contain any protected forest (Table 1), and the 9% of forested 5-km cells that fell partly or wholly within protected areas captured only 15.5% of the global sum of impact scores, compared to a possible maximum of 63.7%. If the proportion of 5-km forest squares that are protected were increased to 17% in line with the target set by the CBD in 2010, the global capture of impact scores could rise from the current 15.5% to a theoretical maximum of 69.5% if new protected areas were sited only in areas with highest impact scores. The 20 IBAs with the highest impact scores are given in Table S2.

### Carbon

**Figure 5:**
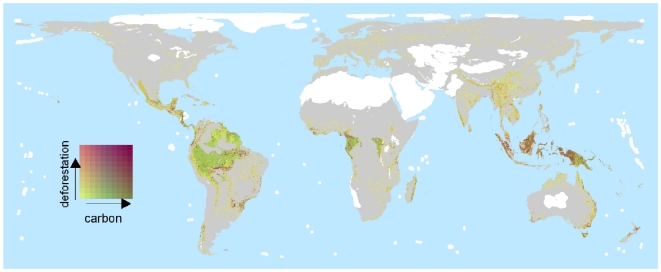
Bivariate plot showing 5-km cell level carbon stocks and rates of forest loss in the top quartile of cells for impact score. Areas in white are non-forested ecoregions or lack data for one or both variables. Areas in grey represent forested or partly forested ecoregions that fall in the lower three quartiles in terms of their maximum impact score.

There was a weak positive association across 5-km cells between impact scores and carbon stocks (Figure 3d), although the pattern varied between biogeographical realms (Figure S3d). Ecoregions with high values (in the upper quartiles for each parameter) of impact score, recent deforestation and carbon stocks include Seram rain forests in Indonesia, Borneo and Sumatra lowland rain forests and Sumatran peat swamp forests in Indo-Malaya, Niger Delta swamp forests (Afrotropics), Madeira-Tapajos moist forest (Palearctic) and Isthmian-Atlantic moist forest in the Neotropics (Figure 5).

## Discussion

### Impact score

Our impact score is a simple metric of conservation value that has been estimated across the globe and is relevant to IUCN Red List criteria A, B and C. It can therefore contribute to the identification of those areas of remaining forest whose loss is likely to have the greatest impact on the conservation status of the world's forest birds. Unlike methods that classify priority sites for conservation in a binary way, the impact score is a spatially explicit continuous variable that can provide insights into variation in conservation importance at a high spatial resolution. As with previous global site prioritisation exercises, it does not incorporate the cost of management (not least because there are no global data on land values), nor is it an analysis of complementarity. At the national scale, which is where practical decisions about delineating and prioritising sites for conservation are made, our score could be incorporated into prioritisation analyses along with data on costs, opportunity and complementarity. The impact score can be recalculated as new data become available on the extent of habitats, as better assessments of species' distributions and altitudinal ranges become available and as taxonomic boundaries change, and can be recalculated at regional or country levels. It could also be used to make absolute comparisons over time within cells and other defined spatial units (e.g. ecoregions), and relative spatial comparisons with similarly derived scores for other taxa.

The reliability of the results depends on the accuracy of the input data (as with all such prioritisation exercises). Ideally our analysis would have been based upon data on the Area Of Occupancy (AOO) of species, but such fine-scale distribution data are available globally for a tiny proportion of forest species. Therefore we took a pragmatic approach and estimated the potential Extent of Suitable Habitat (ESH) for species [24], which reduced commission errors relative to the Extent Of Occurrence (EOO) [13], [25]. Species are unlikely to be distributed evenly across their entire range, meaning that the ESH will usually exceed the Area of Occupancy (AOO). For example, our forest map includes forests that have been degraded to some unknown degree, resulting in the ESH exceeding the AOO for species that cannot tolerate degradation. Other determinants of occupancy and abundance such as hunting pressure cannot be mapped from remote sensing, further increasing discrepancies between ESH and AOO. However, because of the scale at which we report our results and because our score is based on multiple species, we do not think this biases our results, although we acknowledge these limitations [25], [26]. Thus, while our results should prove useful for identifying priority areas for new or expanded IBAs and protected areas or for investing REDD+ resources, defining boundaries of specific sites and prioritising among sites will require local-scale validation.

The degree to which areas with high impact scores for birds capture those of high importance for other taxa cannot yet be assessed, since the extent to which areas of rarity, endemism and risk overlap between major groups is unclear [25], [26], [27]. However, repeating this analysis for other well-mapped taxa (e.g. mammals and amphibians) would be straightforward.

### The distribution of impact scores

The highly skewed frequency distribution of the impact score suggests that protecting a relatively small number of the world's forested areas would yield disproportionate benefits for birds. Tropical islands and mountains often had high scores, with most of the 20 ecoregions containing the highest impact scores falling into one or both of these categories (Table 1). In these areas the high scores are generally a consequence of the importance of the cells for a relatively small number of species with restricted ranges (Figure S2). Previous studies have shown that such areas are often important for restricted-range specie [28], and that areas with many rare or endemic species often have low species richness [27], [29], [30]. The latter is consistent with the weak correlation we found between impact scores and species richness.

### Using the impact score in conservation planning

Most of the world's governments have committed to increase terrestrial protected area coverage to 17% by 2020 [20]. There is therefore a pressing need to identify sites that are the most urgent priorities for protection. This will require consideration of both biodiversity importance (irreplaceability) and degree of threat (vulnerability) [19], [31]. Our impact score is informative for assessing the former, although we recognise that areas with low impact scores may still have high importance for biodiversity (for non-forest species, highly threatened species, significant aggregations of individuals or for non-avian taxa), or for the delivery of ecosystem services. Even though there was no evidence that areas with high impact scores were systematically more or less threatened than other areas (i.e. there was no correlation between impact scores and rates of recent deforestation), overlaying the impact score with recent rates of deforestation and current levels of protection can help identify areas whose addition to the protected area network would yield the greatest benefits to forest birds.

Within areas identified as being of high priority, identification of specific sites for new or expanded protected areas will need to take into account political and socioeconomic realities on the ground. Since IBAs are identified nationally through multi-stakeholder processes as discrete sites that are actual or potential conservation management units, they provide an existing network of sites whose boundaries incorporate such practical considerations. Unprotected (or incompletely protected) IBAs for which formal protection is appropriate and that lie within areas of high irreplaceability (impact score) and high vulnerability (recent deforestation rate) represent some of the most urgent priorities for protected area network expansion if governments are to meet their CBD targets. These include, for example, the Western Ridge and Middle Ridge IBAs in Palau, the Príncipe forest IBA, the São Tomé lowland forest IBA, the Blue Mountains IBA in Jamaica and El Parque Nacional Península de Paria in Venezuela IBA (Table S2). Protection of such IBAs will provide much broader biodiversity benefits, as the IBA network covers about 80% of the extent of Key Biodiversity Area networks in those countries in which of Key Biodiversity Areas for non-avian species have been identified (BirdLife International unpublished data). As well as helping to inform expansion of formal protected area networks, our results should also help to set priorities for other approaches for safeguarding priority sites, including, for example, community management [32]. Furthermore, our results have relevance for REDD+, as this market-based mechanism for mitigating climate change could provide substantial opportunities for biodiversity conservation through the protection of intact forests [21], [23]. Columbia, Indonesia and Panama are the only UN REDD Programme or Partner countries among the 13 countries that contain the 20 IBAs with the highest impact scores. However, REDD+ projects in any of the forests that we have identified as being highly threatened and poorly protected forests as well as having both high carbon values and high impact scores are most likely to deliver benefits for both climate-change mitigation and biodiversity conservation. While Strassburg et al. [33] also found a positive association between regions of high conservation value and high carbon stocks, we pinpoint the most urgent priorities among potential REDD+ opportunities by incorporating additional data on levels of protection and threat (Table 1). Implementation of REDD+ projects in such places has great potential to help safeguard the future of the world's forest bird species and other biodiversity [22], [23], [33].

## Methods

### Bird data and forest cover

Digital distribution maps of the extent of occurrence (EOO) of all bird species were extracted from a recently completed library [34]. These maps were derived from a variety of sources. These include specimen localities obtained from museum data, 587,000 point localities for 6,800 species in BirdLife's Point Locality Database; 5.02 million records for 8,600 species in the Global Biodiversity Information Facility (GBIF), many of which relate to specimen records; observer records documented in BirdLife International's Red Data Books and species factsheets, published literature, survey reports and other unpublished sources; 304,073 records for 7,506 species of documented occurrences in 10,367 Important Bird Areas (extracted from BirdLife's World Bird Database), distribution atlases derived from systematic surveys, distribution maps in field guides and other handbooks, and expert opinion. The digital distribution maps represent the best current estimates of the EOO of all bird species. We analysed the subset of 6,077 species (representing 60.6% of all extant bird species) that are scored in BirdLife International's World Bird Database as having high or medium forest-dependence [10]. Species with high forest-dependence are forest specialists that are characteristic of the interior of undisturbed forest, rarely occupy non-forest habitats, and almost invariably breed within forest; while they may persist in secondary forest and forest patches if their particular ecological requirements are met, they are usually less common in such situations. Species with medium forest-dependence are forest generalists that breed in undisturbed forest but are also regularly found in forest strips, edges and gaps and secondary forest, where they may be commoner than in the interior of intact forest.

For each of these forest-dependent species, altitudinal limits were also extracted from the same source [10] and the EOO maps were clipped by forest cover and altitude to produce maps of the extent of potentially suitable habitat (ESH) within the EOO of each species [13], [35]. The baseline forest cover map used to clip these maps was extracted from GLC2000, and included Global Land Cover Classes 1 to 10 [36]. This included all forested land, from boreal taiga to tropical rainforests. Forest cover mapped from GLC2000 is very similar in extent to that mapped from Modis [37]. Altitudinal data were extracted from a 30 arc second digital elevation model (DEM) produced from the Shuttle Radar Topography Mission data [38]. We adopted a 5-km grid square resolution as a trade-off between spatial explicitness and processing speed, although a spatial resolution of 1 km could be achieved with the data available. A 5-km square was classed as forested if any of its 25 constituent 1-km squares was forested in GLC2000. Nearly two-thirds (62%) of the 5-km cells thereby selected contained at least 50% forest cover. We examined the effects of varying the threshold of forest cover within a cell from 4% (i.e. a single 1-km square) up to 100% (i.e. all 25 1-km squares) for a subset of the data (species endemic to Africa). All species had ESH estimates >0 when a 4% threshold was used (i.e. at least one 5-km square classed as forest), but the proportion of species with ESH estimates of zero increased to 6% with a 20% threshold, 14% with a 60% threshold and 21% with a 100% threshold.

Using these data, we then examined the effect on the impact score for each 5-km cell of using different thresholds (4, 20, 40, 60, 80 and 100%). For each threshold, we log-normalised the impact scores and then regressed them against the scores obtained with a 4% threshold. Although the absolute value of the impact scores increased with increasing threshold (due to fewer cells being used to calculate the values, and despite the loss of 21% of species), the very strong correlations (R^2^ always> = 0.99) indicated that there was very little relative change in cell importance (Table S3).

The minimum and maximum altitudes of each 5-km square were assessed from the DEM and the square was considered to lie within the altitudinal distribution of each species if any part of it fell within the altitudinal limits of that species. Because the majority of 5-km cells contained at least 50% forest cover and altitudinal variation within individual 5-km cells was generally low, the probability that only the non-forested part of a particular square fell within the requisite altitudinal limits was slight, although this might have resulted in a marginal overestimation of ESH. The resulting maps of ESH therefore included, for each forest species, all the 5-km cells within that species' EOO that had partial or complete forest cover in the year 2000 and that fell at least partly within the altitudinal limits of that species.

The ESH maps reduced the EOO extents by 48.2±0.4% but the two were strongly correlated across species (*r* = 0.84). Although there was a significant difference in this reduction between species with high (n = 2609) and medium (n = 3468) forest dependency (χ^2^_1_ = 52.5, P<0.001), the effect size equated to just a 5.3±0.7% difference in EOO reduction. We assessed whether ESH maps reduced the number of omission and commission errors compared to EOO maps [13], [39], using data on the occurrence of globally threatened species at IBAs and the location of these IBAs. Compared to EOO, ESH estimates reduced errors of commission by 19.7% for all species (20.8% and 18.3% for the 317 high and 173 medium forest dependent globally threatened species respectively), while omissions only decreased by 9.5% (9.0% and 10.6% for high and medium dependency respectively). This is consistent with previous studies [13], [39] showing that ESH has fewer commission errors than EOO.

### Calculating the impact score

For each 5-km cells, we estimated an impact score, *s*, as:
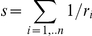
where *r_i_* is the total number of 5-km cells within the estimated distribution (ESH) of the *i*th species and *n* is the total number of species whose ESH includes that 5-km square [16], [17]. Thus, species with restricted ranges contribute more to the overall impact score of each square they occupy than do species with extensive ranges, but all species predicted to occur within a square contribute to its impact score, and, importantly, all species contribute equally at a global scale (i.e. with a value of 1 for 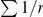). The rationale for this approach is that distribution size is one of the factors identified by IUCN as contributing to extinction risk. Species with restricted ranges are considered to be at high risk of extinction [40], and thresholds of absolute distribution size and rates of decline in distribution are incorporated into the Red List criteria [41]. Furthermore, distribution size is closely correlated with population size across species [42], another factor incorporated into the IUCN Red List criteria. Therefore, the loss of forest in a 5-km square with a high value of *s* will lead to a greater increase in aggregate extinction risk of forest birds globally than loss of forest in a square with a small value of *s*. Our value of *s* can therefore be linked directly to IUCN extinction-risk criteria A, B and C and is comparable between 5-km cells anywhere in the world.

### Recent forest loss

In order to assess the level of threat to ecoregions of high conservation importance, we intersected impact scores with deforestation rates during the period 2000–2005 estimated by Hansen et al. [24]. These data are available at a spatial resolution of 18-km squares, so each forested 5-km square was assigned a value based on the % loss of forest between 2000 and 2005 of the 18-km square within which it wholly or largely fell. Because of differences in the spatial coverage of forests given by GLC2000 and the areas assessed for forest loss in [23], data on forest loss in 2000–2005 were not available for all forested or partly forested ecoregions, particularly those comprising small islands. Consequently, results of analyses relating to forest loss were applicable only to the areas of overlap between GLC2000 and the areas used in [23], which equated to 95% of all forested 5-km cells but excluded many with high impact scores.

### Protected areas and Important Bird Areas (IBAs)

In order to assess the degree of overlap between ecoregions with high biological importance and current conservation investment in the form of protected areas, we intersected impact scores with the global distribution of protected areas from the World Database of Protected Areas [43]. We considered only nationally designated protected areas for which a polygon was included in the database [44]. If any part of the cell overlapped a protected area it was considered protected. We also assessed the overlap of impact scores with Important Bird Areas (IBAs). IBAs are sites representing actual or potential conservation management units, their designation taking into account habitat extent, land use and land ownership [10]. Some 5,198 IBAs (50.7%) for which polygons were available at the time of analysis overlapped with forested areas.

### Carbon stocks

In order to identify areas with potential for safeguarding both carbon and biodiversity, we overlaid impact scores for 5-km cells on a resampled global map of carbon storage derived from Kapos et al. [45], which combines estimates of above- and below-ground biomass [46] and soil carbon storage [47].

### Ecoregion-scale analyses

We report some of our results at the scale of the world's 731 forested or partly forested ecoregions [2], which form biologically distinctive large-scale spatial units. To avoid averaging out small areas of particularly high importance within ecoregions, we report the maximum impact score of any 5-km cell within each ecoregion, although there was tight correlation across ecoregions between maximum and mean scores (Figure S5). Ecoregion-level forest bird species richness was calculated cumulatively across each ecoregion, rather than simply averaged across all cells within that ecoregion.

### Statistical analysis

Generalised additive models were used to assess the relationship between maximum impact score recorded in each ecoregion, the ecoregion-level coverage of protected areas, and (for those ecoregions with data available) recent forest loss. Analysis was undertaken at a 5-km cell scale for carbon storage, owing to the variation in carbon storage across ecoregions. Because global relationships between extinction risk and environmental variables might show strong regional variation [48], analyses were replicated at the level of major biogeographic realms [2]. All spatial data manipulations were undertaken in ArcMap 9.2 (ESRI 2006) and used equal area projections. Statistical analyses were undertaken in *R* 2.12.1 [49]. Means are presented ±1 standard error.

## Supporting Information

Figure S1 **Tif file of the 5 km resolution version of** **Figure 2a** **.**
(7Z) Click here for additional data file.

Figure S2 **Smoothed relationship between impact score in each 5-km cell (vertical axis), estimated bird species richness within the cell (x axis) and the mean log ESH across species in each cell (y axis).**
(DOC)

Figure S3 **Realm-level scatterplots between impact score and (a) overall forest bird species richness, (b) forest loss, 2000–2005, (c) coverage by the protected areas network and (d) carbon stocks.** Fitted lines show GAMs. Data on forest loss and carbon stocks were missing for a number of ecoregions in Oceania, which are omitted from those graphs. Points indicate ecoregions means except in the case of carbon, which is averaged across only forested cells within each ecoregion.
(DOC)

Figure S4 **Mean ± SE impact score in 5-km cells within and outside protected areas.**
(DOC)

Figure S5 **Relationship between mean and maximum values of score in each ecoregion.**
(DOC)

Table S1 Excel file of summary data by ecoregions, showing realm, ranking within realm for maximum impact score, maximum and mean impact scores, species richness of forest birds, recent (2000–2005) deforestation and the percentage of forest within protected areas.
(XLS)

Table S2 The 20 IBAs with the highest maximum impact scores and rates of forest loss (% loss, 2000–2005), with protected area status.
(DOC)

Table S3 Summary of regression between log scores using differing percentage thresholds of 1-km forest cover to define 5-km cells as forested. Regressed against impact score for scores for 1-km (4%) being forest.
(DOC)
