# Supplementary material for: Identifying Priority Areas for Conservation: A Global Assessment for Forest-Dependent Birds
Source: PLoS One. 2011 Dec 19;6(12):e29080. doi: 10.1371/journal.pone.0029080 (PMC3242781; doi:10.1371/journal.pone.0029080)

Figure S2. Smoothed relationship between loss impact score in each 5-km cell (vertical axis), estimated bird species richness within the cell (x axis) and the mean log ESH across species in each cell (y axis).


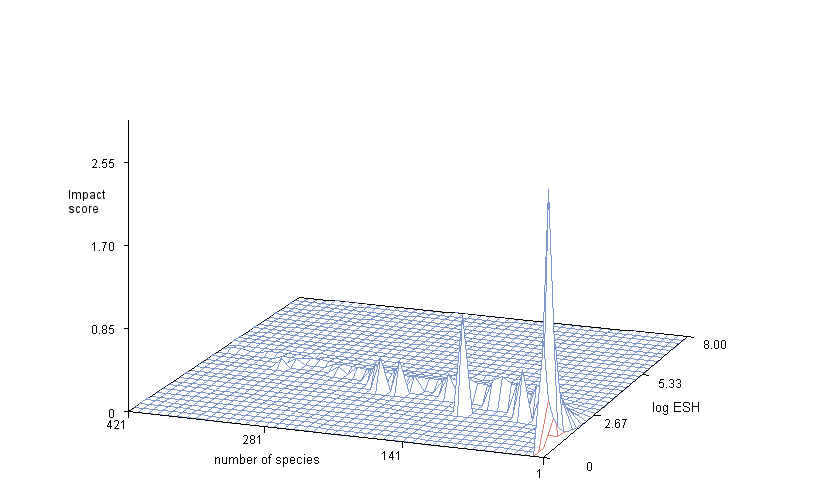

Supplement: Figure S2 — Smoothed relationship between impact score in each 5-km cell (vertical axis), estimated bird species richness within the cell (x axis) and the mean log ESH across species in each cell (y axis). (DOC) [file pone.0029080.s002.doc]
