# Supplementary material for: Identifying Priority Areas for Conservation: A Global Assessment for Forest-Dependent Birds
Source: PLoS One. 2011 Dec 19;6(12):e29080. doi: 10.1371/journal.pone.0029080 (PMC3242781; doi:10.1371/journal.pone.0029080)

Figure S3. Realm-level scatterplots between impact score and (a) overall forest bird species richness, (b) forest loss, 2000-2005, (c) coverage by the protected areas network and (d) carbon stocks. Fitted lines show GAMs. Data on forest loss and carbon stocks were missing for a number of ecoregions in Oceania, which are omitted from those graphs. Points indicate ecoregions means except in the case of carbon, which is averaged across only forested cells within each ecoregion.

S3a


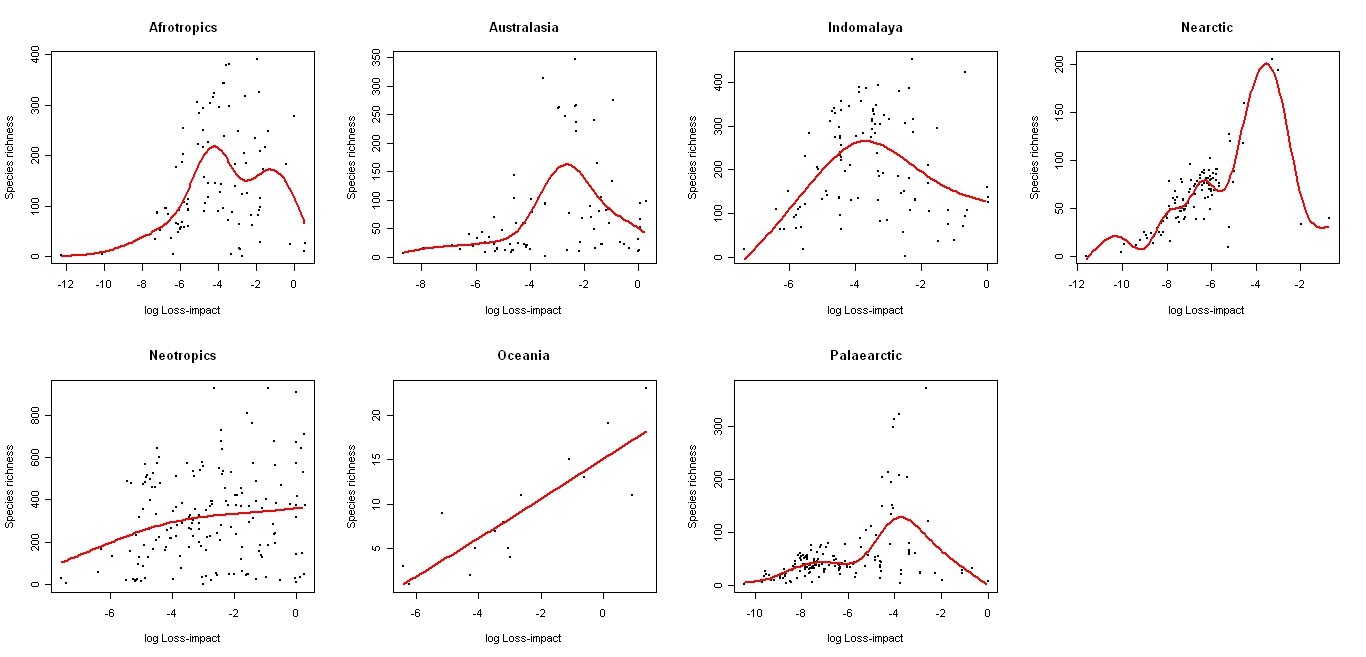


S3b


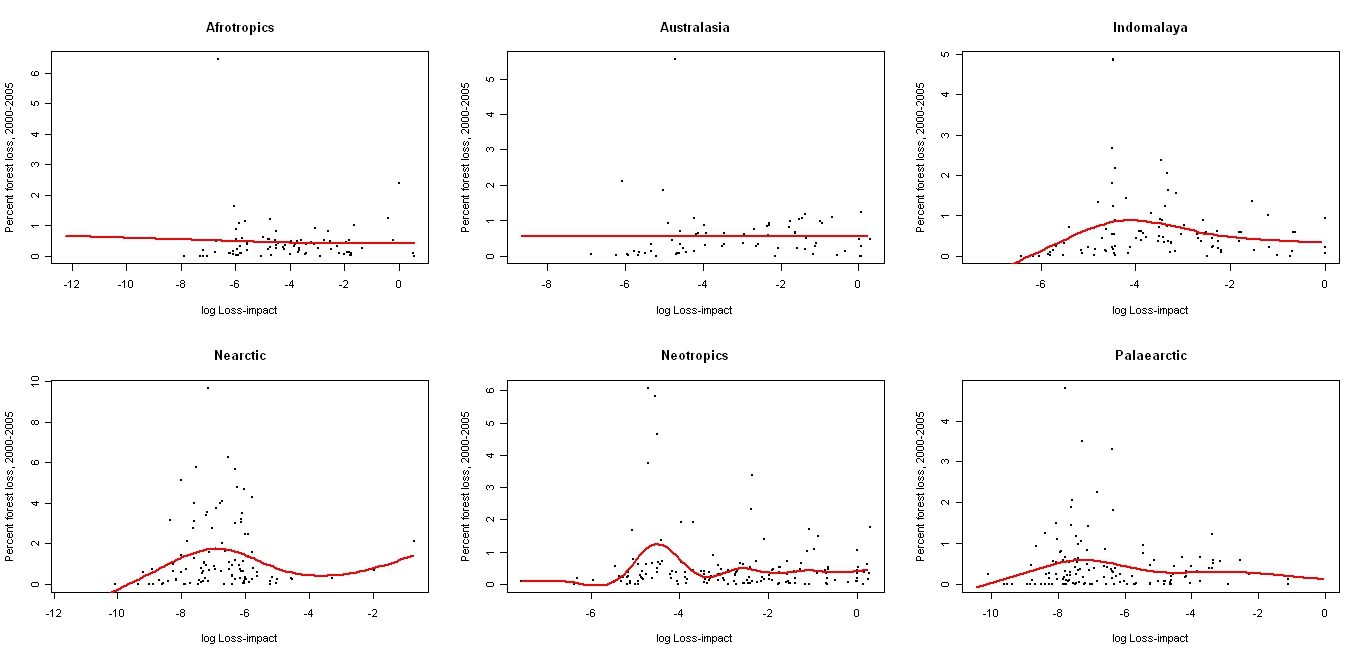


S3c


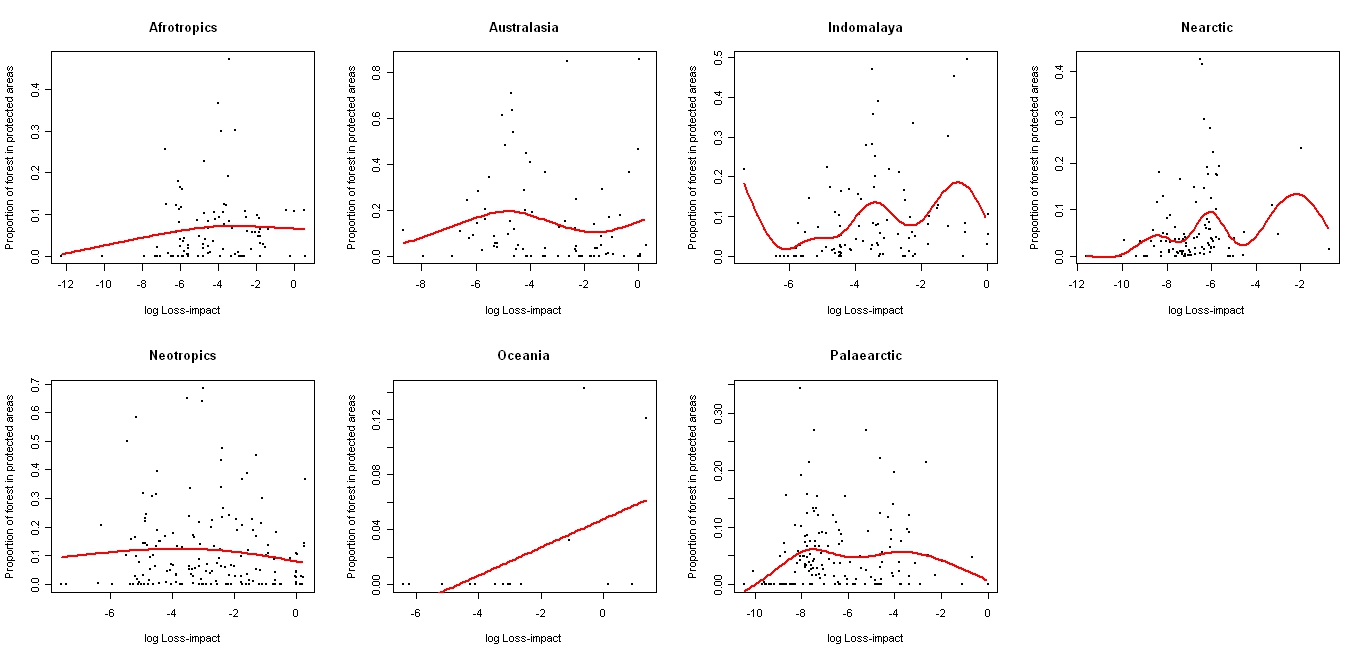


S3d


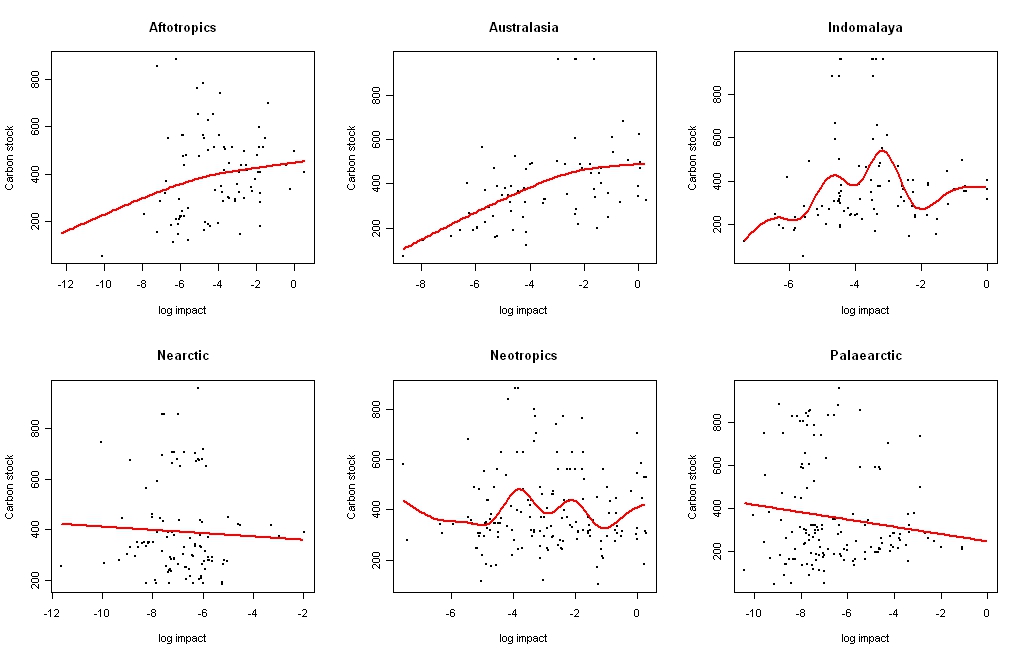

Supplement: Figure S3 — Realm-level scatterplots between impact score and (a) overall forest bird species richness, (b) forest loss, 2000–2005, (c) coverage by the protected areas network and (d) carbon stocks. Fitted lines show GAMs. Data on forest loss and carbon stocks were missing for a number of ecoregions in Oceania, which are omitted from those graphs. Points indicate ecoregions means except in the case of carbon, which is averaged across only forested cells within each ecoregion. (DOC) [file pone.0029080.s003.doc]
