# Supplementary figures and images for: Identifying Priority Areas for Conservation: A Global Assessment for Forest-Dependent Birds
Source: PLoS One. 2011 Dec 19;6(12):e29080. doi: 10.1371/journal.pone.0029080 (PMC3242781; doi:10.1371/journal.pone.0029080)

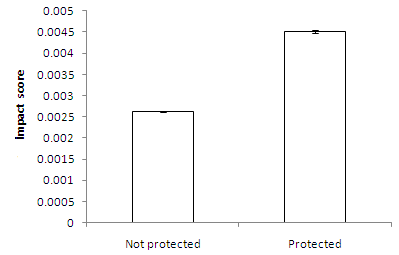


Figure S4. Mean ± SE loss-impact score in 5-km cells within and outside protected areas.

Supplement: Figure S4 — Mean ± SE impact score in 5-km cells within and outside protected areas. (DOC) [file pone.0029080.s004.doc]

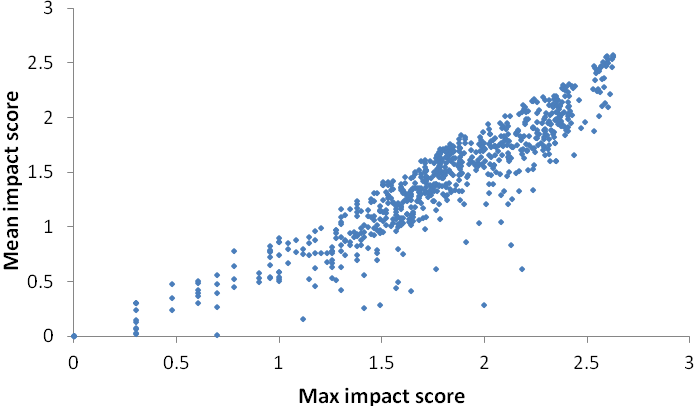


Figure S5 Relationship between mean and maximum values of impact in each ecoregion

Supplement: Figure S5 — Relationship between mean and maximum values of score in each ecoregion. (DOC) [file pone.0029080.s005.doc]
