# Supplementary material for: Identifying Priority Areas for Conservation: A Global Assessment for Forest-Dependent Birds
Source: PLoS One. 2011 Dec 19;6(12):e29080. doi: 10.1371/journal.pone.0029080 (PMC3242781; doi:10.1371/journal.pone.0029080)
Supplement: Table S2 — The 20 IBAs with the highest maximum impact scores and rates of forest loss (% loss, 2000–2005), with protected area status. (DOC) [file pone.0029080.s007.doc]

Table S2. The 20 IBAs with the highest maximum impact scores and rates of forest loss (% loss, 2000-2005), with protected area status

| IBA | Name | Country | Protected | Impact score | Deforestation rate | link |
| --- | --- | --- | --- | --- | --- | --- |
| PW003 | Western Ridge, Babeldaob | Palau | no | 2.5502 | No data | http://www.birdlife.org/datazone/sitefactsheet.php?id=23548 |
| PW002 | Middle Ridge, Babeldaob | Palau | no | 2.5502 | No data | http://www.birdlife.org/datazone/sitefactsheet.php?id=23543 |
| ST004 | Príncipe forests | Sao Tome | no | 1.5388 | No data | http://www.birdlife.org/datazone/sitefactsheet.php?id=6884 |
| VE019 | Parque Nacional Península de Paria | Venezuela | no | 1.3401 | 4.044 | http://www.birdlife.org/datazone/sitefactsheet.php?id=14932 |
| JM013 | Blue Mountains | Jamaica | yes | 1.2952 | 0.514 | http://www.birdlife.org/datazone/sitefactsheet.php?id=18720 |
| ST001 | São Tomé lowland forests | Sao Tome | no | 1.2761 | 0.7455 | http://www.birdlife.org/datazone/sitefactsheet.php?id=6881 |
| PA053 | Darién National Park | Panama | yes | 1.2738 | 0.4765 | http://www.birdlife.org/datazone/sitefactsheet.php?id=19341 |
| CO055 | Cuenca del Río Toche | Columbia | yes | 1.1895 | 0.7455 | http://www.birdlife.org/datazone/sitefactsheet.php?id=14493 |
| FJ11 | Gau Highlands | Fiji | no | 1.1873 | No data | http://www.birdlife.org/datazone/sitefactsheet.php?id=20337 |
| LC002 | Government Forest Reserve | St Lucia | yes | 1.1594 | No data | http://www.birdlife.org/datazone/sitefactsheet.php?id=20575 |
| ID146 | Manupeu-Tanadaru | Indonesia | yes | 1.0766 | 0.514 | <http://www.birdlife.org/datazone/sitefactsheet.php?id=15951> |
| ID201 | Gunung Sibela | Indonesia | yes | 1.0717 | No data | http://www.birdlife.org/datazone/sitefactsheet.php?id=15974 |
| BR188 | Serra dos Órgãos | Brazil | yes | 1.0419 | No data | http://www.birdlife.org/datazone/sitefactsheet.php?id=20215 |
| GQ002 | Basilé Peak National Park | Equatorial Guinea | yes | 1.0176 | No data | <http://www.birdlife.org/datazone/sitefactsheet.php?id=6379> |
| BR042 | Chapada do Araripe | Brazil | yes | 1.0046 | 0.4491 | <http://www.birdlife.org/datazone/sitefactsheet.php?id=20139> |
| TT003 | Northern Range | Trinidad & Tobago | no | 1.0038 | 0.7455 | <http://www.birdlife.org/datazone/sitefactsheet.php?id=20664> |
| BR078 | Alto Rio Juruena | Brazil | no | 1.0025 | 2.5471 | <http://www.birdlife.org/datazone/sitefactsheet.php?id=23424> |
| BR009 | Ilha de Marajó | Brazil | yes | 1.0012 | 0.7802 | http://www.birdlife.org/datazone/sitefactsheet.php?id=22243 |
| PT083 | Laurissilva | Portugal | yes | 1 | No data | http://www.birdlife.org/datazone/sitefactsheet.php?id=19666 |
| VE048 | Caruachi | Venezuela | yes | 1 | 2.0963 | <http://www.birdlife.org/datazone/sitefactsheet.php?id=14988> |
