# Supplementary material for: Identifying Priority Areas for Conservation: A Global Assessment for Forest-Dependent Birds
Source: PLoS One. 2011 Dec 19;6(12):e29080. doi: 10.1371/journal.pone.0029080 (PMC3242781; doi:10.1371/journal.pone.0029080)
Supplement: Table S3 — Summary of regression between log scores using differing percentage thresholds of 1-km forest cover to define 5-km cells as forested. Regressed against impact score for scores for 1-km (4%) being forest. (DOC) [file pone.0029080.s008.doc]

Table S3. Summary of regression between log impact scores using differing percentage thresholds of 1-km forest cover to define 5-km cells as forested. Regressed against impact score for scores for 1-km (4%) being forest.

| **% forest** | **Intercept** | **se** | **Slope** | **se** | **R^2^** |
| --- | --- | --- | --- | --- | --- |
| **20** | 0.39 | 0.0005 | 1.02 | 0.0001 | 0.99 |
| **40** | 0.76 | 0.0007 | 1.04 | 0.0001 | 0.99 |
| **60** | 1.14 | 0.0010 | 1.08 | 0.0002 | 0.99 |
| **80** | 1.14 | 0.0010 | 1.08 | 0.0002 | 0.99 |
| **100** | 1.13 | 0.0010 | 1.07 | 0.0002 | 0.99 |
|  |  |  |  |  |  |
